# Predictors of Serum Chlorinated Pesticide Concentrations among Prepubertal Russian Boys

**DOI:** 10.1289/ehp.1306480

**Published:** 2013-08-16

**Authors:** Thuy Lam, Paige L. Williams, Jane S. Burns, Oleg Sergeyev, Susan A. Korrick, Mary M. Lee, Linda S. Birnbaum, Boris Revich, Larisa M. Altshul, Donald G. Patterson, Wayman E. Turner, Russ Hauser

**Affiliations:** 1 Environmental and Occupational Medicine and Epidemiology Program, Department of Environmental Health, and; 2 Department of Biostatistics, Harvard School of Public Health, Boston, Massachusetts, USA; 3 Samara State Medical University, Department of Physical Education and Health, Samara, Russia; 4 Chapaevsk Medical Association, Chapaevsk, Samara Region, Russia; 5 Channing Division of Network Medicine, Department of Medicine, Brigham and Women’s Hospital, Harvard Medical School, Boston, Massachusetts, USA; 6 Pediatric Endocrine Division, Department of Pediatrics and Cell Biology, University of Massachusetts Medical School, Worcester, Massachusetts, USA; 7 National Cancer Institute, and; 8 National Institute for Environmental Health Sciences, National Institutes of Health, Department of Health and Human Services, Research Triangle Park, North Carolina, USA; 9 Institute for Forecasting, Russian Academy of Sciences, Moscow, Russia; 10 Exposure, Epidemiology, and Risk Program, Department of Environmental Health, Harvard School of Public Health, Boston, Massachusetts, USA; 11 Environmental Health and Engineering, Inc., Needham, Massachussetts, USA; 12 EnviroSolutions Consulting Inc., Auburn, Georgia, USA; 13 Axys Analytical Solutions, Sidney, BC, Canada; 14 Exponent, Inc., Maynard, Massachusetts, USA; 15 Centers for Disease Control and Prevention, Atlanta, Georgia, USA

## Abstract

Background: Few studies have evaluated predictors of childhood exposure to organochlorine pesticides (OCPs), a class of lipophilic persistent chemicals.

Objectives: Our goal was to identify predictors of serum OCP concentrations—hexachlorobenzene (HCB), β-hexachlorocyclohexane (β-HCH), and *p,p*-dichlorodiphenyldichloroethylene (*p,p*´-DDE)—among boys in Chapaevsk, Russia.

Methods: Between 2003 and 2005, 499 boys 8–9 years of age were recruited in a prospective cohort. The initial study visit included a physical examination; blood collection; health, lifestyle, and food-frequency questionnaires; and determination of residential distance from a local factory complex that produced HCB and β-HCH. Fasting serum samples were analyzed for OCPs at the U.S. Centers for Disease Control and Prevention. General linear regression models were used to identify predictors of the boys’ serum HCB, β-HCH, and *p,p´*-DDE concentrations.

Results: Among 355 boys with OCP measurements, median serum HCB, β-HCH, and *p,p´*-DDE concentrations were 158, 167, and 284 ng/g lipid, respectively. Lower body mass index, longer breastfeeding duration, and local dairy consumption were associated with higher concentrations of OCPs. Boys who lived < 2 km from the factory complex had 64% (95% CI: 37, 96) and 57% (95% CI: 32, 87) higher mean HCB and β-HCH concentrations, respectively, than boys who lived ≥ 5 km away. Living > 3 years in Chapaevsk predicted higher β-HCH concentrations, and having parents who lacked a high school education predicted higher *p,p´*-DDE concentrations.

Conclusions: Among this cohort of prepubertal Russian boys, predictors of serum OCPs included consumption of local dairy products, longer local residence, and residential proximity to the local factory complex.

Citation: Lam T, Williams PL, Burns JS, Sergeyev O, Korrick SA, Lee MM, Birnbaum LS, Revich B, Altshul LM, Patterson DG Jr, Turner WE, Hauser R. 2013. Predictors of serum chlorinated pesticide concentrations among prepubertal Russian boys. Environ Health Perspect 121:1372–1377; http://dx.doi.org/10.1289/ehp.1306480

## Introduction

Persistent, lipid-soluble organochlorine pesticides (OCPs) such as hexachlorobenzene (HCB), β-hexachlorocyclohexane (β-HCH), dichlorodiphenyltrichlorethane (DDT), and its primary metabolite *p,p*´-dichlorodiphenyl-dichloroethylene (*p,p*´-DDE) are ubiquitous in the environment. Although these insecticides and fungicides were banned in the 1970s (United States) and in the 1980s (Russia) (Barber et al. 2005; Breivik et al. 1999; Jaga and Dharmani 2003), DDT is still used in some countries to control malaria and yellow fever (Jaga and Dharmani 2003), and HCB and β-HCH are generated as by-products during the manufacture of other chlorinated chemicals (Courtney 1979; Jung et al. 1997). In addition to biomagnifying up the food chain, these compounds have long half-lives in both the environment and the body, ranging from years to decades, and persist long after use has ceased (Barber et al. 2005; Courtney 1979; Jaga and Dharmani 2003; Jung et al. 1997).

Human exposure typically occurs through diet (e.g., fatty fish, dairy products, meats, poultry) and less commonly through inhalation or dermal absorption (Darnerud et al. 2006; Gasull et al. 2011). OCPs are stored in adipose tissue, concentrated in breast milk (Rogan et al. 1986), and can be passed from mother to child via transplacental transfer or breastfeeding, a primary route of exposure for children (Rogan et al. 1986; Sala et al. 2001). Compared with adults, young children have disproportionately elevated exposures to pesticides because they have higher ventilatory rates and food consumption relative to body weight (National Research Council 1993) and greater hand-to-mouth transfer of contaminated soil and dust. Moreover, the ability to metabolize, detoxify, and excrete pesticides may be reduced in young children (Landrigan et al. 2003).

HCB, β-HCH, and *p,p*´-DDE have been associated with adverse health effects in animal studies, including neurodevelopmental toxicity (Courtney 1979; Ecobichon et al. 1990), cancer [Courtney 1979; International Agency for Research on Cancer (IARC) 1987, 1991], decreased male reproductive performance (e.g., mating index), and testicular abnormalities (Courtney 1979; Gralla et al. 1977; Gray et al. 2001; Van Velsen et al. 1986). Among humans, there is evidence of neurodevelopmental toxicity following chronic exposure (reviewed by Ecobichon et al. 1990). Understanding the predictors of OCP exposure can improve child health and inform public health recommendations and policies to reduce childhood exposure. To date, some studies have evaluated predictors of childhood exposure to OCPs (Barr et al. 2006; Den Hond et al. 2009; Gallo et al. 2011; Karmaus et al. 2001; Lu et al. 2010; Petrik et al. 2006).

We assessed predictors of serum HCB, β-HCH, and *p,p*´-DDE concentrations among children residing in Chapaevsk, Russia, a city of approximately 72,000 people located 950 km southeast of Moscow that has a history of environmental organochlorine compound contamination. In the 1930s, a complex of factories began producing chemical agents for military use (e.g., mustard gas, lewisite). From 1967 to 1987, production focused on organochlorine chemicals including HCB, HCH, and its derivatives (α-HCH, β-HCH, and γ-HCH) (Akhmedkanov et al. 2002). However, DDT was never produced (Sergeyev et al. 2008). By 2003, production of all chemicals ceased. Environmental contamination by HCB and β-HCH may have resulted from improper disposal, storage of hazardous waste from factories, or environmental release of organochlorine by-products of the manufacturing process (Shelepchikov et al. 2008).

## Methods

*Study population*. The Russian Children’s Study is an ongoing prospective cohort study of 499 prepubertal boys in Chapaevsk, Russia. Briefly, 623 boys 8–9 years of age were identified from the town-wide health insurance system. Of these, 572 were eligible and 516 agreed to participate (recruitment rate of 90%), although 17 boys were subsequently excluded because they were orphans (precluding collection of residential history and other information). The first 144 9-year-old boys enrolled did not have OCPs measured, leaving 355 boys with serum OCP measurements. The study was approved by the Human Studies Institutional Review Boards of the Chapaevsk Medical Association, Harvard School of Public Health, University of Massachusetts Medical School, and Brigham and Women’s Hospital. The parent/guardian of each child gave informed consent, and the children signed assent forms before participation.

At study entry, eligible boys had a physical examination and a fasting blood sample collected. A study nurse administered health and lifestyle questionnaires to the parent/guardian that included questions concerning the child’s birth history, medical history, and physical activity; parental occupational and educational background; family medical history and residential history; and household income. The child’s current residence was identified using an electronic map.

*Physical examination*. At the initial visit, height and weight measurements were obtained according to a written protocol. Height in stocking feet was measured to the nearest 0.1 cm using a stadiometer. Weight was measured to the nearest 100 g with a metric scale. Body mass index (BMI) was calculated as kilograms divided by height in meters squared.

*Blood sample analysis*. Fasting blood samples collected at study entry were centrifuged, and the serum was aliquoted and stored at –35°C until shipment on dry ice to the U.S. Centers for Disease Control and Prevention for analysis. Five OCPs (HCB, β-HCH, *p,p´*-DDE, aldrin, and mirex) were measured in the 355 boys. The samples, including method blank and quality control samples, were spiked with ^13^C_12_-labeled pesticides, extracted by a C18 solid-phase extraction (SPE) followed by a multicolumn automated cleanup and enrichment procedure using either large-volume (Turner et al. 1997) or small-volume SPE (Sjödin et al. 2004). High-resolution mass spectrometry in selective ion monitoring was used to analyze the samples (Barr et al. 2003). Because aldrin and mirex were below the limit of detection (LOD) in almost all (> 95%) participants (median aldrin LOD, 0.40 ng/g lipid; median mirex LOD, 4.5 ng/g lipid), we limited our analysis to HCB, β-HCH, and *p,p´*-DDE, all of which were above the LOD in all samples (median HCB LOD, 4.1 ng/g lipid; median β-HCH LOD, 4.5 ng/g lipid; median *p,p*´-DDE LOD, 5.6 ng/g lipid). The analytical coefficients of variation for individual OCPs in quality assurance/quality control samples ranged between 10% and 15% over the course of the study. All OCP concentrations were expressed on a per-lipid basis. Total serum lipid content was determined from enzymatic measurements of total cholesterol and triglycerides (Phillips et al. 1989).

*Distance information*. The straight-line distance from each participant’s residence to the primary factory complex was calculated using ArcView GIS 3.0 (ESRI, Redlands, CA, USA) by the Moscow Ecological Analytical Center.

*Food frequency questionnaire (FFQ)*. Study nurses administered a validated Russian Institute of Nutrition FFQ that was modified to assess local food consumption (Hauser et al. 2005). The FFQ included questions on the usual frequency of consumption and portion sizes (classified based on photographs) of > 70 food items consumed during the previous year (Martinchik et al. 1998). The participating boy and his parent/guardian completed the FFQ together.

*Statistical analysis*. We used Spearman correlations to assess relationships among OCPs. We used linear regression models to identify predictors of the serum concentration of each OCP. To improve normality and reduce the influence of outliers, serum OCP concentrations were log_10_-transformed. Potential predictors were identified using *a priori* knowledge from the literature. All covariates (except food consumption) with *p* ≤ 0.20 in bivariate analyses were included in an initial multivariable linear regression model for each OCP that was subsequently reduced to include predictors with *p* ≤ 0.10 only. In addition, breastfeeding duration was retained in all models because it is a known route of exposure for children, and there was a significant linear trend (*p* ≤ 0.05) in the concentration of all three OCPs over categories of breastfeeding duration. We categorized breastfeeding duration as none, 1–13 weeks, and > 13 weeks to limit the potential influence of a small number of observations with a long duration of breastfeeding. To account for nonlinearity, BMI was grouped according to World Health Organization (WHO) child growth standards: underweight (> 1 SD below the mean), normal, and overweight/obese (> 1 SD above the mean) (de Onis et al. 2007). We modeled other continuous predictors as simple continuous variables and as categorical variables, and examined point estimates and compared the goodness-of-fit of each model based on adjusted coefficient of determination (*R*^2^) values to determine the most appropriate coding.

Individual food items were categorized into groups (eggs, dairy products, poultry, non-poultry meats, fish, and fruits and vegetables), and the usual frequency and portion size (grams/year) of each category was determined. In addition, we estimated consumption of local foods in each food category, and included each local food category [in tertiles (low, medium, or high) or dichotomized as any vs. none when < 50% reported eating local foods] in multivariable models while simultaneously adjusting for total consumption of each food category (in tertiles). Local foods reflect possible local contamination, whereas total foods are adjusted in models to reflect background levels. Because of the specific interest in dietary sources of OCP concentrations, each food group was considered separately as well as simultaneously, adjusting for other factors.

For ease of interpretation, regression coefficients are presented as the estimated percent change in the serum concentration of each OCP with a 1-unit increase in exposure (for continuous variables) or relative to the reference category (for categorical variables) (10^β^, where β is the regression coefficient for a given predictor), holding all other variables constant. We also estimated adjusted mean concentrations of each OCP according to residential distance from the factory using least-square means, adjusted for all other predictors in the final model for each OCP. Additionally, we performed sensitivity analyses using log_10_-transformed whole-weight serum OCP concentrations adjusted for total lipids instead of direct lipid–adjusted OCPs in the final model. A *p*-value of < 0.05 was considered statistically significant. Tests for trend were performed by modeling categorical variables as an ordinal variable using integer values (0, 1, 2). All data analyses were performed using SAS version 9.2 (SAS Institute Inc., Cary, NC, USA).

## Results

*Study population*. Characteristics of the 355 boys are shown in Table 1. At study entry, 84% of boys were 8 years old, 17% were overweight/obese, and 25% were underweight (de Onis et al. 2007). Among boys with and without serum OCP measurements (*n* = 355 vs. 144), there were no significant differences in height, weight, BMI *z*-scores, birth, or family characteristics, but there was a significant difference in household income (44% vs. 26% of families in the highest income category, respectively). Also, more boys with measured serum OCP concentrations had fathers who were employed at the factory complex compared with those without OCP measurements (14% vs. 9%).

**Table 1:** Characteristics of 8- to 9-year-old ­participants in the Russian Children’s Study at study entry (*n* = 355).

Characteristic	Total boys
Growth measurements
Height (cm)	129 ± 6.04
Weight (kg)	26.7 ± 5.53
BMI	15.9 ± 2.33
WHO height *z*-score	0.13 ± 1.01
WHO BMI *z*-score	–0.16 ± 1.31
Birth and neonatal history
Birth weight (kg)	3.3 ± 0.53
Gestational age (weeks)	39.0 ± 1.81
Duration of breastfeeding (weeks)	
None	46 (13)
1–13	143 (40)
> 13	160 (45)
Boys’ dietary consumption of any local foods
Dairy	151 (42)
Poultry	29 (8)
Non-poultry meats	20 (6)
Fish	76 (21)
Eggs	54 (15)
Fruits and vegetables	341 (96)
Parental and residential characteristics
Duration of Chapaevsk residence (years)	
< 3	101 (28)
3 to < 6	71 (20)
6 to < 8	94 (26)
≥ 8	87 (25)
Any household smoking during pregnancy	59 (17)
Mother ≤ 25 years old at son’s birth	248 (70)
Maximum parental education	
High school or less	30 (8)
Junior college/technical school	201 (57)
University/postgraduate training	122 (34)
Household income ($US/month)	
< 175	110 (31)
175–250	89 (25)
> 250	155 (44)
Father employed at factory complex	50 (14)
Residential distance to factory complex (km)	
< 2	65 (18)
2 to < 5	159 (45)
≥ 5	131 (37)
Data are mean ± SD or *n* (%) unless stated otherwise. Missing: Birth weight, *n *= 1 (0.3%); Gestational age, *n *= 2 (1%); Duration of breastfeeding, *n *= 6 (2%); Local dairy consumption, *n *= 3 (1%); Local poultry consumption, *n *= 3 (1%); Local non-poultry meat consumption, *n *= 3 (1%); Local fish consumption, *n *= 3 (1%); Local egg consumption, *n *= 5 (1%); Local fruit and vegetable consumption, *n *= 3 (1%); Duration of Chapaevsk residence, *n *= 2 (1%); Any household smoking during pregnancy, *n *= 5 (1%); Mother ≤ 25 years old at son’s birth, *n *= 3 (1%); Maximum parental education, *n *= 2 (1%); Household income, *n *= 1 (0.3%); Father employed at factory, *n *= 20 (6%). Percentages may not total 100% due to rounding.	

*Distribution of serum HCB,* β*-HCH,* p,p´*-DDE.* The medians (25th, 75th percentiles) for serum HCB, β-HCH, and *p,p*´-DDE concentrations were 158 (107, 246), 167 (112, 270), and 284 (187, 492) ng/g lipid, respectively (Table 2). Median *p,p*´-DDE concentrations were about three times higher than concentrations previously reported for adolescents in the United States and Belgium, whereas HCB concentrations were about 7–12 times higher (Table 3) (Den Hond et al. 2011; Patterson et al. 2009). The median β-HCH concentration previously reported for 12- to 19-year-old U.S. adolescents (below the LOD of 7.8 ng/g lipid) (Patterson et al. 2009) was at least 20 times lower than the median concentration in our study population of Russian boys. Spearman correlations between the OCPs were *r* = 0.61 for β-HCH and *p,p*´-DDE, *r* = 0.54 for β-HCH and HCB, and *r* = 0.34 for HCB and *p,p*´-DDE.

**Table 2:** Distribution of measured OCPs (ng/g lipid) among 8- to 9-year-old boys enrolled in the Russian Children’s Study (*n* = 355).^*a*^

OCP	*n*	Percentile						
		Minimum	10th	25th	50th (median)	75th	90th	Maximum
HCB	355	32	80	107	158	246	364	2,660
β-HCH	355	39	81	112	167	270	412	2,860
*p,p*´-DDE	355	49	122	187	284	492	835	9,370
^***a***^No values < LOD.								

**Table 3:** Median OCP concentrations (ng/g lipid) in 8- to 9-year-old boys in the Russian Children’s Study compared with other pediatric studies.

Country	Year	*n*	Age range (years)	Population	HCB	β-HCH	*p,p*´-DDE
Russia (current study)	2003–2005	355	8–9	Boys	158	167	284
USA (NHANES)^*a*^	2003–2004	588	12–19	Boys and girls	13.4	< LOD	93.6
Belgium^*b*^	2003–2004	1,679	14–15	Boys	22.8	—	104
Faroe Islands^*c*^	1986–1987	788	14	Boys and girls	—	—	467
Slovakia^*d*^ (contaminated Michalovce district)	2001	216	8–10	Boys and girls	79.6	—	344
Abbreviations: —, OCP not measured; NHANES, National Health and Nutrition Examination Survey. LOD = 7.8 ng/g lipid. ^***a***^Patterson et al. (2009). ^***b***^Den Hond et al. (2011). ^***c***^Barr et al. (2006). ^***d***^Petrik et al. (2006).							

*Predictors of serum HCB,* β*-HCH,* p,p´*-DDE.* BMI and residential distance from the factory complex were the strongest predictors, explaining 18% and 23% of the variability in the serum concentrations of HCB and β-HCH, respectively. All other covariates combined explained an additional 3% (HCB) and 13% (β-HCH) of the variability. For *p,p*´-DDE, BMI and breastfeeding duration combined explained 23% of the variability, all other model covariates explained an additional 7%.

**Figure 1:**
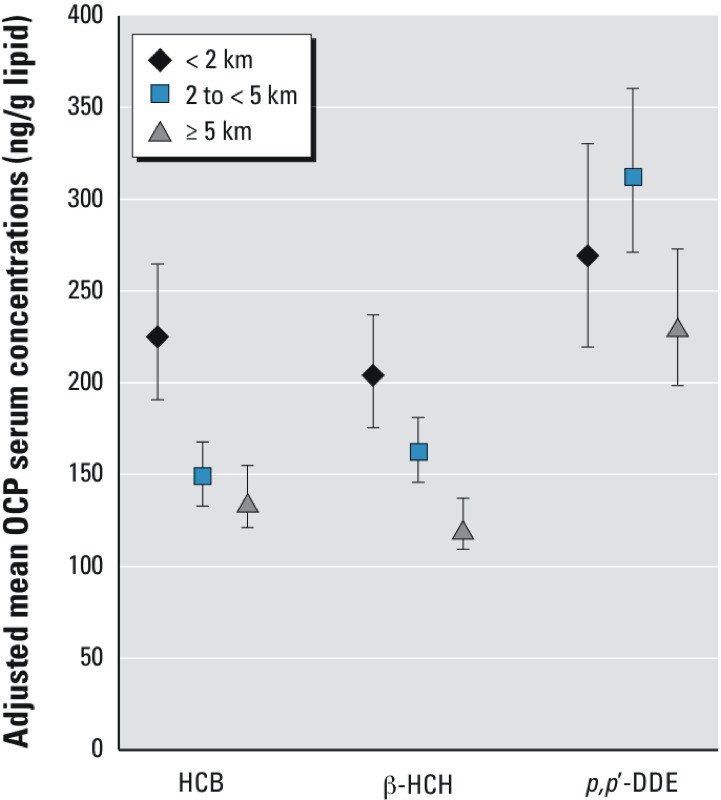
Adjusted mean OCP serum concentrations of Russian prepubertal boys in relation to residential distance from factory complex. Adjusted means (95% CIs) calculated using least-square means adjusted for all other covariates in Table 4.

Boys breastfed > 13 weeks had 16% (95% CI: –5, 41%), 63% (95% CI: 35, 96%), and 81% (95% CI: 43, 128%) higher predicted mean serum HCB, β-HCH, and *p,p*´-DDE concentrations, respectively, than non-breastfed boys, with a significant linear trend over increasing categories of breastfeeding for all three OCPs (Table 4). BMI was also a significant predictor of all three OCPs, with the highest mean concentrations among boys who were classified as underweight, and the lowest among boys who were overweight or obese. Living near the factory complex predicted increased serum concentrations of HCB (9.3%; 95% CI: –4.9, 25.6% and 64%; 95% CI: 37, 96% for 2 to < 5 km and < 2 km compared with ≥ 5 km, respectively), and β-HCH (33.7%; 95% CI: 17.1, 52.7% and 57.1%; 95% CI: 32, 87%, respectively.) Living 2 to < 5 km from the factory was a significant predictor of serum *p,p*´-DDE concentrations (35.6%; 95% CI: 15.1, 59.8%), whereas predicted concentrations were increased but lower for boys living < 2 km from the factory (16.7%; 95% CI: –5.2, 43.7%) (Table 4). Specifically, for boys living < 2 km from the factory complex, these estimated increases correspond to mean HCB, β-HCH, and *p,p*´-DDE serum concentrations of 223 ng/g lipid (95% CI: 191, 260 ng/g lipid), 208 ng/g lipid (95% CI: 181, 240 ng/g lipid), and 284 ng/g lipid (95% CI: 233, 346 ng/g lipid), respectively, adjusted for the final model covariates for each OCP (Figure 1).

**Table 4:** Final multivariable predictor models for serum concentrations of organochlorine pesticides based on linear regression models.

	HCB (*n *= 346)		β-HCH (*n *= 327)		*p,p*´-DDE (*n *= 346)	
	Estimated % change in pesticide (95% CI)^*a*^	*p*-Value^*b*^	Estimated % change in pesticide (95% CI)^*a*^	*p*-Value^*b*^	Estimated % change in pesticide (95% CI)^*a*^	*p*-Value^*b*^
WHO BMI *z*-score categories
Underweight	28.3 (10.6, 48.8)	0.001	18.6 (3.1, 36.5)	0.02	9.0 (–8.5, 29.8)	0.34
Normal	Reference		Reference		Reference
Overweight/obese	–36.1 (–46.3, –23.9)	< 0.001	–44.1 (–52.5, –34.2)	< 0.001	–51.0 (–60.0, –39.9)	< 0.001
*p*-Value**for trend		< 0.001		< 0.001		< 0.001
Breastfeeding duration (weeks)
None	Reference		Reference		Reference
1–13	2.0 (–16.3, 24.4)	0.84	13.2 (–6.4, 36.8)	0.20	8.0 (–14.4, 36.4)	0.52
> 13	15.9 (–4.8, 41.0)	0.14	62.8 (35.0, 96.3)	< 0.001	81.0 (43.3, 128.4)	< 0.001
*p*-Value**for trend		0.05		< 0.001		< 0.001
Residential distance from factory complex (km)
< 2	63.8 (37.0, 95.9)	< 0.001	57.1 (31.8, 87.2)	< 0.001	16.7 (–5.2, 43.7)	0.15
2 to < 5	9.3 (–4.9, 25.6)	0.21	33.7 (17.1, 52.7)	< 0.001	35.6 (15.1, 59.8)	< 0.001
≥ 5	Reference		Reference		Reference
*p*-Value**for trend		< 0.001		< 0.001		0.03
Local dairy consumption	14.4 (0.6, 30.1)	0.04	20.6 (6.9, 36.1)	0.003	17.5 (1.0, 36.7)	0.04
Total dairy consumption^*c*^
Low	Reference		Reference		Reference
Medium	–2.7 (–16.6, 13.5)	0.72	–2.9 (–16.1, 12.3)	0.69	–7.5 (–22.7, 10.8)	0.40
High	–0.7 (–15.0, 16.1)	0.93	–2.2 (–15.5, 13.2)	0.76	–10.7 (–25.8, 7.4)	0.23
*p*-Value**for trend		0.93		0.76		0.23
Duration of Chapaevsk residence (years)
< 3	—	—	Reference		—	—
3 to < 6	—	—	28.3 (7.9, 52.5)	0.005	—	—
6 to < 8	—	—	28.0 (9.1, 50.2)	0.003	—	—
≥ 8	—	—	28.0 (9.0, 50.4)	0.003	—	—
*p*-Value**for trend	—	—		0.003	—	—
Father worked at factory complex	—	—	16.0 (–2.6, 38.0)	0.10	—	—
Maximum parental education	—	—	—	—
High school or less	—	—	—	—	57.2 (16.9, 111.4)	0.003
Junior college/technical school	—	—	—	—	8.1 (–8.0, 27.1)	0.34
University/postgraduate	—	—	—	—	Reference
*p*-Value**for trend	—	—	—	—		0.01
Total model *R*^2^	0.21		0.36		0.30
—, variables were not retained in final models. Final models include predictors with *p* ≤ 0.10. ^***a***^Estimated change in pesticide concentration based on β parameter estimates for predicting log_10_ lipid-adjusted concentrations and then calculating 10^β^.^***b***^Wald statistic. ^***c***^Total dairy consumption is included in final models to reflect background levels.						

Any local dairy consumption (vs. none) predicted higher HCB, β-HCH, and *p,p*´-DDE serum concentrations of 14% (95% CI: 0.6, 30%), 21% (95% CI: 7, 36%), and 18% (95% CI: 1, 37%), respectively, even with adjustment for total dairy consumption (which was not a significant predictor of any of the OCPs) (Table 4). Although any local poultry consumption predicted higher HCB (*p* = 0.09) and total egg consumption predicted higher β-HCH (*p* = 0.09), only local dairy consumption was a significant predictor when poultry, egg, and dairy consumption were modeled simultaneously (data not shown).

Factors that predicted higher concentrations of only one of the OCPs were having parents who had a high school education or less (57% higher serum *p,p*´-DDE relative to boys whose parents had university/postgraduate training; 95% CI: 17, 111%), living in Chapaevsk for ≥ 3 years (higher β-HCH concentrations compared with those living < 3 years in Chapaevsk, trend *p* = 0.003) and having a father employed at the factory complex (higher β-HCH concentrations, *p* = 0.10).

The percent of variation (*R*^2^) explained by the predictors in each final OCP model ranged from 0.21 (for the model of HCB) to 0.36 (for β-HCH). Sensitivity analyses of predictors for whole weight of log_10_-transformed serum OCPs adjusted for total lipids were consistent with predictions of lipid-adjusted serum OCPs (see Supplemental Material, Table S1).

## Discussion

In the present study, we measured serum OCP concentrations and identified several demographic, lifestyle, and environmental predictors among boys living in Chapaevsk, Russia, a town contaminated by previous industrial activity. These results complement a publication describing predictors of dioxins and polychlorinated biphenyls (PCBs) among these boys (Burns et al. 2009). Despite the young age of our cohort (8–9 years), concentrations of OCPs were similar to or higher than concentrations reported for somewhat older pediatric populations (range, 8–19 years) in the United States and Europe (Barr et al. 2006; Den Hond et al. 2011; Patterson et al. 2009; Petrik et al. 2006).

Consistent with other studies of persistent organic pollutants in this cohort and other populations (Burns et al. 2009; Den Hond et al. 2009; Gallo et al. 2011; Humblet et al. 2010; Karmaus et al. 2001), lower BMI predicted higher serum OCP concentrations. This finding may be attributable to a smaller volume of distribution in boys with lower BMI, resulting in higher serum concentrations (Wolff et al. 2005).

Breastfeeding is a known route of early life exposure to lipophilic persistent compounds (Rogan et al. 1986). Consistent with other studies (Barr et al. 2006; Den Hond et al. 2009; Gallo et al. 2011; Karmaus et al. 2001), longer breastfeeding duration (> 13 weeks) predicted higher OCP concentrations. Although breastfeeding in our cohort ended years before OCP measurement, childhood concentrations of lipophilic compounds track closely with breastfeeding exposure in infancy (Patandin et al. 1999).

Residential distance from the primary factory may provide insight on chemical-specific pathways of exposure in this area. Specifically, living < 2 km from the complex was associated with higher serum HCB and β-HCH concentrations, which is consistent with these compounds having been manufactured at the factory as a source of exposure. However, serum *p,p*´-DDE levels were highest for boys living 2 to < 5 km from the factory and only moderately and nonsignificantly elevated among boys living within 2 km. DDT, the parent compound for *p,p*´-DDE, was not manufactured at the complex, and other exposure sources likely contributed to the boys’ *p,p*´-DDE levels.

Duration of residence in Chapaevsk and father’s prior employment at the factory were positive predictors of β-HCH. We hypothesized that longer residence in Chapaevsk would be associated with higher exposure to and bioaccumulation of both β-HCH and HCB, because both were produced locally. Therefore, it is unclear why duration of residence was a significant predictor of β-HCH but not HCB. Similarly, fathers’ prior occupation at the factory was not a significant predictor of HCB, despite the same potential for exposure from residues on the fathers’ work clothing, boots, tools, or skin (Lu et al. 2000). Mother’s employment at the factory did not predict any of the OCPs, but only 5% of mothers reported previous employment at the factory, limiting power to detect a statistically significant association.

Non-occupational exposure to HCB, β-HCH, and *p,p*´-DDE is primarily dietary (Darnerud et al. 2006; Gasull et al. 2011); therefore, we expected consumption of local foods high in fat, such as dairy and fish, to predict higher serum concentrations.However, local dairy consumption was the only significant predictor of all three OCPs. Studies of OCP exposure and local diet among children have been limited to two assessments concerned about local environmental contamination (Den Hond et al. 2009; Gallo et al. 2011), that did not find an association with local dairy. Dairy consumption has been associated with serum OCP concentrations in several adult populations, although none differentiated whether dairy foods were from local sources (Arrebola et al. 2009, 2012; Lee et al. 2007). Although previous studies among children reported associations between serum OCPs and consumption of local fish (Gallo et al. 2011) and consumption of fatty meats and vegetables (Den Hond et al. 2009), these foods were not significant predictors in our cohort.

We previously reported that consumption of most local foods predicted higher serum dioxins and PCBs in the same study cohort (Burns et al. 2009), consistent with findings reported for other study populations concerned about environmental contamination (Choi et al. 2006; Gallo et al. 2011; Schecter et al. 2003). It is unclear why local food consumption, apart from dairy, was associated with dioxin-like compounds but not serum OCP concentrations in our study cohort.

One limitation of our diet analyses is the inability to assess consumption of specific types of fish or fat content. We had only father’s reported employment history with no independent verification, and therefore can only speculate on the association observed with β-HCH. Although we attempted to evaluate many potential determinants of these exposures (e.g., parental education), there were probably other predictors that we could not assess.

A major strength of this study is the large sample size of young boys with serum HCB, β-HCH, and *p,p*´-DDE measurements. For all three OCPs, all serum concentrations were above the limit of detection with wide ranges of concentrations. Detailed dietary information, including local food consumption, as well as calculated residential distance from the factory complex, was also available. In this context, this study contributes to understanding of determinants of serum OCP levels among children, and in particular, highlights the potential importance of local risk factors for exposure.

## Conclusion

Our findings suggest that contamination from the local factory may be an important source of HCB and β-HCH exposure for boys in Chapaevsk. Residential distance from the primary factory was a significant predictor of serum HCB and β-HCH, both of which were manufactured at the complex. Father’s past employment at the factory and longer residence in Chapaevsk also predicted higher serum β-HCH, and local dairy consumption predicted higher serum concentrations of all three OCPs; these results add further support to local environmental contamination, at least partly from the factory, as a source of exposure. Consistent with other studies, longer breastfeeding duration and lower BMI predicted higher serum OCPs.

Our findings provide insight on determinants of OCP exposure, which may lead to local monitoring and continuation of remediation measures (e.g., soil removal) to reduce childhood and community exposure. Although our findings suggest that local dairy consumption and longer breastfeeding duration are primary determinants of OCP exposure, it would be premature to recommend reduced intake of local dairy foods or reduced breastfeeding without fully understanding the exposure pathway and the risk–benefit trade-offs from such a recommendation. It is important to keep in mind that these local food products were central to the children’s diet in this region, and that breastfeeding has well-established benefits. Recommendations to prevent childhood exposure include environmental cleanup of contaminated areas, regulatory enforcement of safe practices for industrial waste disposal and emissions control, and preferential consumption, when available, of foods produced in noncontaminated areas.

## Supplemental Material

(283 KB) PDF
