# Supplementary material for: Predictors of Serum Chlorinated Pesticide Concentrations among Prepubertal Russian Boys
Source: Environ Health Perspect. 2013 Aug 16;121(11-12):1372–7. doi: 10.1289/ehp.1306480 (PMC3855511; doi:10.1289/ehp.1306480)
Supplement: (283 KB) PDF [file ehp.1306480.s001.pdf]

Environmental Health Perspectives doi:10.1289/ehp.1306480

Supplemental Material

Predictors of Serum Chlorinated Pesticide Concentrations among Pre-Pubertal Russian Boys

Thuy Lam, Paige L. Williams, Jane S. Burns, Oleg Sergeyev, Susan A. Korrick, Mary M. Lee, Linda S. Birnbaum, Boris Revich, Larisa M. Altshul, Donald G. Patterson Jr., Wayman E. Turner, and Russ Hauser

Supplemental Material, Table S1. Final multivariable predictor models for serum concentrations of whole weight of organochlorine pesticides

	HCB (n=346)		β-HCH (n=327)		*p,p'*-DDE (n=346)	
	Estimated % Change in Pesticide (95% CI)[a]	p-value[b]	Estimated % Change in Pesticide (95% CI)[a]	p-value[b]	Estimated % Change in Pesticide (95% CI)[a]	p-value[b]
**Total lipids**	0.08 (0.002, 0.16)	0.05	0.09 (0.01, 0.16)	0.02	0.09 (-0.01, 0.18)	0.08
**WHO BMI Z categories**						
Underweight	30.1 (12.2, 50.8)	0.001	20.0 (4.4, 38.0)	0.01	10.9 (-6.9, 32.1)	0.25
Normal	REF		REF		REF	
Overweight/Obese	-32.8 (-43.7, -19.9)	<0.001	-41.3 (-50.2, -30.7)	<0.001	-48.6 (-58.2, -36.8)	<0.001
p for trend		<0.001		<0.001		<0.001
**Breastfeeding duration**						
None	REF		REF		REF	
1-13 wks	0.46 (-17.5, 22.4)	0.96	12.0 (-7.2, 35.1)	0.24	5.9 (-16.1, 33.7)	0.63
>13 wks	15.9 (-4.6, 40.8)	0.14	63.5 (35.8, 96.8)	<0.001	79.6 (42.4, 126.6)	<0.001
p for trend		0.05		<0.001		<0.001
**Residential distance from factory complex**						
<2 km	67.5 (40.2, 100.1)	<0.001	60.5 (34.7, 91.1)	<0.001	19.2 (-3.2, 46.8)	0.10
2 to <5 km	8.7 (-5.4, 24.7)	0.24	32.8 (16.5, 51.5)	<0.001	35.2 (14.8, 59.2)	<0.001
≥5 km	REF		REF		REF	
p for trend		<0.001		<0.001		0.02
**Local dairy consumption**	12.8 (-0.75, 28.2)	0.06	18.7 (5.3, 33.9)	0.01	16.3 (-0.02, 35.2)	0.05
**Total dairy consumption**[c]						
Low	REF		REF		REF	
Medium	-1.4 (-15.4, 14.9)	0.86	-1.9 (-15.1, 13.3)	0.79	-6.5 (-21.9, 12.0)	0.47
High	1.2 (-13.4, 18.3)	0.88	-0.63 (-14.1, 14.9)	0.93	-9.3 (-24.6, 9.1)	0.30
p for trend		0.88		0.93		0.30
**Duration of Chapaevsk Residence**						
<3 yrs	---	---	REF		---	---

	HCB (n=346)		β-HCH (n=327)		*p,p'*-DDE (n=346)	
	Estimated % Change in Pesticide (95% CI)[a]	p-value[b]	Estimated % Change in Pesticide (95% CI)[a]	p-value[b]	Estimated % Change in Pesticide (95% CI)[a]	p-value[b]
3 to <6 yrs	---	---	30.2 (9.7, 54.6)	0.003	---	---
6 to <8 yrs	---	---	27.9 (9.1, 49.8)	0.002	---	---
≥8 yrs	---	---	26.0 (7.4, 47.9)	0.005	---	---
p for trend	---	---		0.01	---	---
**Father worked at factory complex**	---	---	15.2 (-3.1, 37.0)	0.11	---	---
**Maximum parental education**	---	---	---	---		
High school or less	---	---	---	---	49.6 (11.0, 101.6)	0.01
Junior college/Technical school	---	---	---	---	6.9 (-9.0, 25.6)	0.42
University/Post-Graduate	---	---	---	---	REF	
p for trend	---	---	---	---		0.03
Total model R-square	0.21		0.36		0.29	

[a]Estimated change in pesticide concentration based on β parameter estimates for predicting log (base 10) lipid-adjusted concentrations and then calculating 10^{β}

[b] p-value is from Wald statistic

[c]Total dairy consumption is included in final models to reflect background levels.

Final models include predictors with p<0.10
